# Identification of Faecal Maternal Semiochemicals in Swine (*Sus scrofa*) and their Effects on Weaned Piglets

**DOI:** 10.1038/s41598-020-62280-9

**Published:** 2020-03-24

**Authors:** Edgar O. Aviles-Rosa, Kaz Surowiec, John McGlone

**Affiliations:** 1 Laboratory of Animal Behavior, Physiology and Welfare, Department of Animal and Food Sciences, Collage of Agricultural Sciences and Natural Resourses, Texas Tech University, 1248 Indiana Ave, Lubbock, TX 79415 USA; 2 Department of Chemistry and Biochemistry, College of Arts and Sciences, Texas Tech Universtity, 2500 Broadway, Lubbock, TX 79409 USA

**Keywords:** Chemical ecology, Animal behaviour

## Abstract

Piglets are attracted to maternal faeces early in life. Thus, the aim of this study was to identify faecal maternal semiochemicals that attract piglets and evaluate their effects on piglets at weaning. Faecal samples were collected from eight sows during gestation and lactation. Faecal semiochemicals were extracted and identified using solid phase extraction and GC/MS. A total of 26 volatiles were present in lactating and gestating sow faeces. Sows secreted no unique semiochemical after farrowing. However, the concentration of skatole and myristic acid were 2.68 and 1.13 times higher after farrowing. A free-choice preference assessment showed that piglets had a preference for a feeder sprayed with a solution containing skatole and myristic acid. No preference was found when feeders were sprayed with skatole and myristic acid individually. The application of skatole and myristic acid to the feeders of weaned pigs significantly reduced piglet aggression by 30% and tended to increase feeding behaviour by 35% the first 24 h post-weaning. These results suggest that skatole and myristic acid might be acting as a multicomponent maternal signal that attracts piglets and has a calming effect at weaning.

## Introduction

Several behavioural studies have demonstrated that piglets are attracted to their mothers’ faeces as early as 12 hours after birth and can even discriminate between maternal and non-maternal faeces^1,2^. Moreover, it has been shown that piglets are attracted to lactating sow faeces when tested against non-lactating sow faeces^2^. This suggests that during lactation, sows might be secreting maternal faecal semiochemicals that attract piglets. At first glance, the biological benefits of swine faecal semiochemicals may seem unobvious. Notwithstanding, maternal faecal pheromones have been reported in other animals. For instance, like piglets, rat pups are attracted to maternal faeces and both species consume maternal faeces^3,4^. Rat dams secrete a maternal faecal pheromone (deoxycholic acid) from the 14^th^ to the 27^th^ day of lactation that attracts pups and promotes coprophagy^5^. By eating maternal faeces, rat pups obtain bile acids that aid the myelination process and guard the guts against pathogenic bacteria^5^.

Although not fully studied, scientists believed that coprophagy helps piglets to prevent anaemia^3,6–11^. In a previous study, we found that piglets deprived of maternal faeces for their first seven days of age had lower growth rate than those exposed to maternal faeces early in life^12^. In this study, we speculated that, as in rats, maternal semiochemicals might be attracting piglets and inducing coprophagy^12^. However, to date, changes in the nutritional value of lactating sow faeces and in its volatile profile has not been evaluated. Since rat pups and piglets exhibit coprophagy behaviour and this behaviour is beneficial to both species, we hypothesize that, as in rats, maternal semiochemicals could be responsible for piglet preference towards maternal faeces and of inducing coprophagy. Hence, the objectives of this study were: (1) The identification of swine maternal faecal semiochemicals that could be attracting piglets. (2) Evaluate changes in lactating sow faeces nutrient content. (3) Evaluate piglets preference for faecal maternal semiochemicals and their effects on piglet behaviour and performance when sprayed on the weaning environment. For this, we compared the nutrient content and volatile profile of gestating and lactating sow faeces and conducted behavioural test with possible candidate semiochemicals.

## Results

### Faeces nutritional value

Even when sows were given the same amount of feed, gestating sows had higher feed intake than lactating sows. Faecal dry matter, acid detergent fibre, ashes, true protein (TP), Ca, Mg, K, Zn, Cu, and Mn changed with sow reproductive state. During lactation, faecal dry matter, ash, TP, Mg, Zn, and Cu increased whereas the acid detergent fibre, Ca, and K decreased (Table 1). No differences in faecal crude protein (CP), neutral detergent fibre, fat, P, Na, Fe, Mn, and Mo were observed.

**Table 1:** Sow faeces nutrient content.

Nutrient	Period		SE^a^	*P* value^b^
	Gestation (N = 8)	Lactation (N = 8)		
FI, kg/d	5.28	3.71	0.34	0.01
DM, %	33.35	37.51	0.55	<0.01
CP, %	21.86	21.10	0.43	0.29
TP, %	12.64	16.33	0.98	<0.01
ADF, %	8.81	7.95	0.62	0.04
NDF, %	23.54	21.38	1.41	0.14
Fat, %	4.20	3.99	0.30	0.73
Ash, %	28.63	31.74	0.60	<0.01
Ca, %	6.84	6.36	0.17	<0.01
P, %	4.62	4.76	0.15	0.21
Mg, %	1.30	1.46	0.04	<0.01
K %	0.82	0.64	0.06	<0.01
Na, %	0.17	0.14	0.02	0.23
Fe, ppm	4237	4038	137	0.26
Zn, ppm	1084	1257	40.2	<0.01
Cu, ppm	153	244	11.8	<0.01
Mn, ppm	832	989	29.2	<0.01
Mo, ppm	5.56	5.17	0.26	0.07

^a^Largest Standard error of least squares means.

^b^Significance level of the effect of period (gestation or lactation).

FI - Feed intake;

DM – Dry matter.

CP - crude protein, nitrogen % *6.25.

TP- True Protein as determine by the Bicinchoninic acid assay.

ADF- Acid detergent fibre.

NDF- neutral detergent fibre.

### Identification of maternal semiochemicals

**Figure 1:**
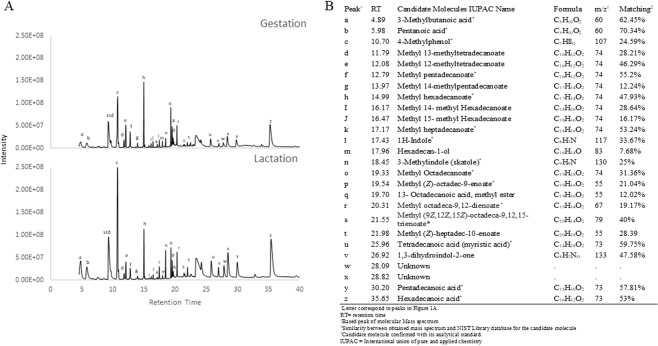
Representative chromatogram of gestating and lactating sow faeces volatiles (**A**) and the names of each volatile present in sow faeces. (**B**) Twenty-six volatiles were present in sow faeces during gestation and lactation. No specific semiochemical was secreted during gestation or lactation. std = internal standard peak.

Figure 1A shows a representative chromatogram of gestating and lactating sow faecal extract. The present study identified 26 volatile organic compounds (VOCs) in sow faeces during gestation and lactation (Fig. 1B). Of these, 15 were confirmed using analytical standards mass spectrum and retention time. The number of volatiles present in faeces was not different during lactation or gestation. Neither gestating nor lactating sow faecal matter had unique volatiles. Nevertheless, the peak area ratio of seven volatiles (Table 2) significantly changed after farrowing. At the beginning of lactation, the peak area ratio of skatole (*P* = < 0.01), myristic acid (*P* = 0.01), and two unknown molecules (*P* = 0.02 and *P* = 0.01) increased whereas the area ratio of methyl (*Z*)-octadec-9-enoate (*P* = 0.001), 13-octadecanoic acid-methyl ester (*P* = 0.03), and methyl octadeca-9,12-dienoate (*P* = 0.01) significantly decreased. Using calibration curves and correcting for faecal dry matter differences between periods, the faecal concentration of candidate semiochemicals whose peak area ratio significantly changed were estimated. Table 3 shows the 95% confidence interval of the estimated concentration of these analytes in wet faeces and on dry matter basis. On wet faeces, skatole and myristic acid faecal concentration significantly increased whereas methyl octadeca-9,12-dienoate concentration significantly decreased during lactation. However, after correcting for faecal dry matter differences between periods, only skatole and methyl octadeca-9,12-dienoate concentrations were significantly different between periods (*P* < 0.05).

**Table 2:** Faecal volatiles peak area ratio.

Candidate Molecule IUPAC name	Gestation (N = 8)	Lactation (N = 8)	SE^a^	*P* value^b^
3-Methylbutanoic acid*	0.07	0.09	0.03	0.57
Pentanoic acid*	0.05	0.05	0.03	0.99
4-Methylphenol*	0.95	0.93	0.06	0.77
Methyl 13-Methyltetradecanoate	0.07	0.09	0.01	0.11
Methyl 12-Methyltetradecanoate	0.19	0.25	0.03	0.19
Methyl pentadecanoate*	0.14	0.15	0.02	0.58
Methyl 14-Methylpentadecanoate	0.03	0.04	0.01	0.42
Methyl hexadecanoate*	0.52	0.38	0.07	0.10
Methyl 14- Methyl Hexadecanoate	0.02	0.03	0.01	0.10
Methyl 15- Methyl Hexadecanoate	0.03	0.04	0.01	0.10
Methyl heptadecanoate*	0.01	0.01	<0.01	0.40
1H-Indole*	0.07	0.04	0.01	0.06
Hexadecan-1-ol	0.03	0.03	0.01	0.95
3-Methylindole (skatole)*	0.09	0.23	0.02	<0.01
Methyl Octadecanoate*	0.22	0.20	0.02	0.52
Methyl (*Z*)-octadec-9-enoate*	0.25	0.13	0.03	0.01
13- Octadecanoic acid, Methyl ester	0.10	0.06	0.01	0.03
Methyl octadeca-9,12-dienoate*	0.24	0.15	0.02	0.01
Methyl (9*Z*,12*Z*,15*Z*)-octadeca-9,12,15-trienoate*	0.01	0.02	<0.01	0.47
Methyl (*Z*)-heptadec-10-enoate	0.09	0.09	0.01	0.92
Tetradecanoic acid (myristic acid)^*^	0.17	0.24	0.02	0.01
1,3-dihydroindol-2-one	0.07	0.06	0.01	0.24
Unknown	0.09	0.23	0.05	0.02
Unknown	0.27	0.43	0.04	0.01
Pentadecanoic acid*	0.15	0.23	0.04	0.12
Hexadecanoic acid*	0.89	0.91	0.07	0.841

^a^SE standard error of the difference.

^b^Significance level of period (Gestation or Lactation) effect.

IUPAC - International union of pure and applied chemistry.

*Candidate molecule confirmed with its analytical standard.

**Table 3:** Candidate semiochemicals estimated concentration.

Candidate analyte		Gestation CI*	Lactation CI*	*P* value
Skatole	^a^µg/g	7.68–15.69	27.29–35.30	<0.01
	DM basis	23.84–46.25	72.44–94.85	<0.01
Methyl octadeca-9,12-dienoate	^a^µg/g	16.17–25.13	9.14–18.10	0.03
	DM basis	48.23–76.23	22.70–50.69	0.01
Myristic acid	^a^µg/g	62.52–69.96	71.23–78.67	<0.01
	DM basis	186.93–210.46	188.58–212.11	0.83

CI* - Lower and upper 95% confident interval.

DM basis - µg/g of dry matter.

^a^Wet (fresh) faeces basis.

### Preference test

Table 4 shows the overall preference index (PI) and the percentage of time piglets spent interacting with feeders sprayed with a solution containing skatole, myristic acid or both semiochemicals. The overall percentage of time piglets interacted with the feeders (*P* = 0.02) and the preference index (*P* = 0.05) showed that piglets preferred the feeder sprayed with a solution containing skatole and myristic acid when compared to a control. No preference or aversion was found when skatole and myristic acid were sprayed individually. Although piglets interacted with the feeders, no feed was consumed during the trials. Thus, feed intake assessment was not possible.

**Table 4:** Maternal semiochemicals preference assessment.

Comparison		Molecule	Control	SE*	N	*P* value
Both^a^ *vs* Control	PI	0.54	0.46	0.02	9	0.05
	%	1.02	0.83	0.11		0.02
Skatole *vs* Control	PI	0.51	0.50	0.02	8	0.74
	%	0.54	0.50	0.11		0.64
Myristic acid *vs* Control	PI	0.47	0.53	0.03	8	0.30
	%	0.48	0.61	0.08		0.22

^a^skatole and myristic acid.

*Largest standard error of the means.

Control – feeder sprayed with mineral oil.

Molecule - feeder sprayed with skatole, myristic acid or both.

### Weaning trial

**Figure 2:**
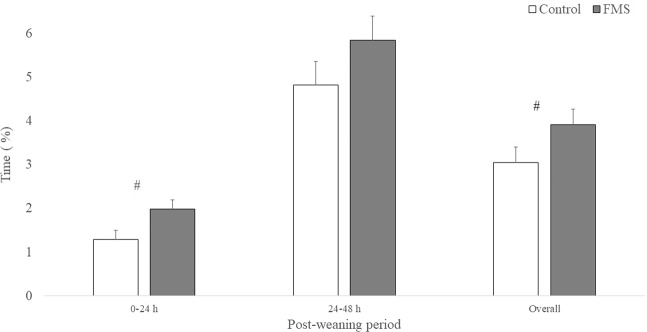
Percentage of time + standard error control (n = 12) and faecal maternal semiochemicals (FMS; n = 12) treated pigs spent feeding during the first 48 hours post-weaning. # = a tendency to be statistically different (0.05 < *P* < 0.10).

**Figure 3:**
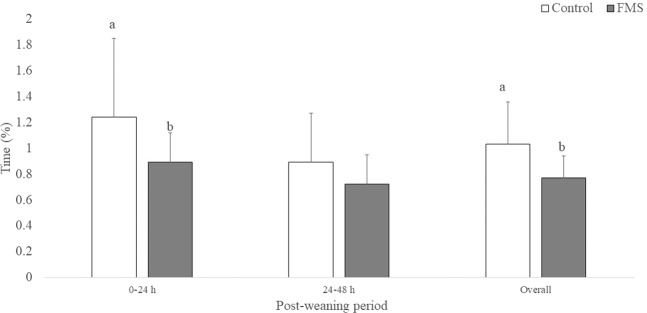
Percentage of time + 95% confidence interval control (n = 12) and faecal maternal semiochemicals (FMS; n = 12) treated engaged in aggressive behaviours during the first 48 hours post-weaning. Bars with different superscript within period are significantly different from each other due to a treatment effect (*P* < 0.05).

Figures 2 and 3 present a summary of piglets’ feeding and aggressive behaviours the first 48 hours post-weaning, respectively. Pigs exposed to the faecal maternal semiochemicals (FMS) spent significantly less time engaged in aggressive behaviours during the first 24 hours post-weaning (*P* = 0.03). Faecal maternal semiochemicals tended to increase piglet feeding behaviour the first 24 hours post-weaning (*P* = 0.09). No differences in feeding or aggressive behaviours were observed from 24 to 48 h post-weaning. Over the entire 48-hour period, FMS reduced pig aggression (*P* = 0.04) and tended to increase feeding behaviour (*P* = 0.08). The interaction between treatment and time was not statistically significant for feeding and aggressive behaviours. The time effect was significant only for feeding behaviour where piglets spent more time eating in the 24–48 h period compared to the the 0–24 h period (5.33 ± 0.37% *vs* 1.64 ± 0.14%; *P* = < 0.01). There was no treatment effect on piglet body weight, average daily gain (0.35 ± 0.02 kg *vs* 0.34 ± 0.02 kg), and average daily feed intake (0.52 ± 0.03 kg *vs* 0.55 ± 0.03 kg) during the first 28 days post-weaning. There was a significant treatment effect on piglets Gain:Feed ratio. Control pigs had better Gain:Feed ratio (0.67 ± 0.02 *vs* 0.62 ± 0.02; *P* = 0.02) compared to FMS treated pigs. The treatment by time interaction was not statistically significant for all performance variables measured.

## Discussion

Changes in sow faecal volatile profile and nutrient content were evaluated during gestation and lactation to identify candidate maternal faecal semiochemicals and determine the nutritional value of lactating sow faeces. Sows reduced their feed intake during the first week of lactation. This was expected since a reduction in feed intake is usually observed after farrowing^13^. During lactation, sow faeces had higher dry matter content. This could be a result of the reduction in feed intake since low feed intake reduces passage rate and increases faecal dry matter content^14^. The increase in water requirements sows have during lactating^15^ promotes intestinal water absorption and could also increase faecal dry matter. During lactation the faecal concentration of certain nutrients and minerals changed. The reduction in Ca^2+^ and K^+^ faecal concentrations during lactation might be the result of the high levels of Ca^2+^ and K^+^ excreted in the milk^16^. Lactating sow faeces contained higher concentration of true protein (TP). In this study, TP was defined as the amount of biological protein present in sow faeces that reacted to the Bicinchoninic acid assay (BCA), not the nitrogen percentage in the faeces. This is a more reliable measure since it does not quantify non-protein nitrogen in faeces. The increase of TP during lactation can be a result of the reduction in crude protein ileal digestibility that has been observed during lactation^17,18^ and the low feed intake sows had during the first days in lactation^19,20^. In addition, the increased cortisol level associated with the farrowing process^21^ could promote gut protein synthesis^22^ thus leading to higher faecal protein content. The observed increase in faecal dry matter and the low faecal output during lactation could explain why some minerals and nutrients were in higher concentrations during lactation since undigested nutrients were clustered in a smaller volume. Further studies are required to evaluate the effect of sow physiological status on nutrients and minerals digestibility. We will not expand our discussion in this regard since this topic is out of the scope of this study. Based on these results, consumption of maternal faeces will result in the ingestion of minerals and nutrients that could be beneficial to piglets. This could explain why deprivation of maternal faeces reduced piglet performance^12^.

A total of 26 VOCs were found in sow faecal matter by GC/MS analysis. The VOCs present in sow faeces were consistent with the ones previously reported in the literature. For instance, pentanoic acid, 3-methylbutanoic acid, 4-methylphenol, 3-methylindole, and indole have been found in growing pig faeces and in piggeries’ air, manure, slurry, and dust^23–29^. Fourteen different fatty acid methyl ether (FAME) were found in sow faeces. Of these, only methyl-hexadecanoate and methyl-octadecanoate have been previously reported in piggeries^26^. Most of the FAMEs found in sow faeces are part of the whole-cell composition of swine faecal coliforms^30,31^. Thus, it could be possible that FAMEs present in sow faecal extract came from bacterial membrane degradation during the extraction process. Further studies are needed to determine the origin of these FAMEs. Likewise, long chain saturated fatty acids such as tetradecanoic (myristic), pentadecanoic and hexadecenoic acids were found in sow faeces. Long chain fatty acids are commonly present in faeces since they are feed constituents. The faecal presence of these fatty acids has been used as an indicator of hind-gut bacterial activity^32^.

Olfaction plays a critical role in piglet maternal recognition^1,2,33^. It has been speculated that lactating sows emit faecal semiochemicals that attract piglets^2^. The present study did not find a unique faecal semiochemical secreted by sows during lactation that might be acting as a chemical signal and thus attracting piglets and inducing coprophagy. Notwithstanding, the peak area ratio of seven molecules changed with sow physiological status. The peak area ratio and the estimated concentration of skatole and myristic acid significantly increased during lactation whereas the peak area ratio and the concentration of methyl octadeca-9,12-dienoate decreased during lactation. After correcting for faecal dry matter differences between periods, only the concentration of skatole and methyl linoleate significantly changed during lactation. Thus, the changes in dry matter content might explain the increase in concentration of myristic acid. In addition to these analytes, the peak area ratio of two unknown analytes significantly increased during lactation. These unknown analytes were not successfully identified in this study since candidate molecules analytical standards mass spectrum and retention time did not matched with the ones obtained in the samples.

Herein was found that skatole faecal concentration increased by almost three-fold during lactation. Skatole is commonly found in swine manure and, together with androstenone it causes the boar taint^34–37^. Of all intestinal microbes, only *Clostridium spp*. and *Lactobacillus spp*. can degrade tryptophan or indole acetic acid into skatole^35–37^.

In sows, skatole faecal concentration changes with the oestrus cycle^38,39^. During oestrus, sows skatole faecal concentration increased from 12 to 50 µg/g DM^38^. In this study, skatole concentration was correlated with sow feed intake and oestradiol and progesterone levels during oestrus^38^. Moreover, in sows, high levels of growth hormone, insulin-like growth factor one (IGF-1), and glucocorticoids increased skatole faecal excretion because these hormones promote intestinal mucosal cell proliferation thus providing more substrate to the intestinal microflora^39^.

In this study, skatole faecal concentration was estimated to increase from 11.69 ± 1.86 µg/g during gestation to 31.29 ± 1.86 µg/g during lactation. When expressed on dry matter basis, the estimated skatole faecal concentration in this study was higher than the one reported by Claus *et al*.^38^. This difference can be due to the physiological state of the animals used in both studies (pregnant and lactating *vs* non-pregnant sows) and the extraction procedure. Notwithstanding, in both studies, the increase in skatole concentration was close to three- and four-fold of magnitude during lactation and oestrus, respectively. The same hormonal and nutritional changes that increased skatole faecal concentration during oestrus^38^ might explain the increment in skatole faecal concentration after farrowing. For instance, after farrowing and in oestrus, sow progesterone levels decrease and sows tend to reduce their feed intake^40^. In addition, early after farrowing, plasma cortisol, prolactin, growth hormone, and oxytocin increase^21,41^. All these physiological changes might increase mucosal turnover^39^ promoting skatole synthesis by microbes. The observed increase of true protein in sow faeces after farrowing support this theory. Hormonal changes after farrowing could increase mucosal turnover providing more substrate to the bacteria thus increasing skatole faecal concentration.

Myristic acid concentration also increased during lactation. This could be due to the increase in faecal dry matter content observed after farrowing since no difference was found when the concentration was expressed on dry matter basis. To the author’s knowledge, this change has not been reported previously in the literature. Surprisingly, myristic acid has been found in the secretion of sebaceous glands of sows, mares, bitches, cows, ewes, queens, and does^42^. In mammals, a combination of fatty acids, including myristic acid, act as a maternal pheromone guiding newborn of the species mentioned above to the mammary gland^42^. In pigs, myristic acid is also present in maternal fluids such as amniotic fluid, colostrum, and milk^43^. The fact that reproductive hormones regulate skatole faecal concentration and that myristic acid is one of the constituents of domestic animal maternal pheromones supports the idea that these two molecules might be acting as maternal semiochemicals in lactating sow faeces. Since piglets have a very acute sense of smell, it is possible that by detecting changes in the concentration of these semiochemicals, they can discriminate between lactating and gestating sow faeces.

The behavioural tests conducted using skatole and myristic acid showed that piglets were attracted to a feeder sprayed with skatole and myristic acid compared to a control feeder. However, piglets were not attracted to the individual analytes. The presence of a maternal faecal semiochemical have been suggested in a previous study where piglets showed a preference for lactating sow faeces when tested against non-lactating sow faeces^2^. Since the major volatile difference between lactating and non-lactating sow faeces was the increased in concentration of skatole and myristic acid, we hypothesized that the increased in concentration of these analytes, might be acting as a multicomponent chemical signal in lactating sow faeces.

Skatole has an attractant effect in different species. For instance, the mosquito *Culex quinquefasciatus* uses skatole as an organic signal to select oviposition site^44,45^. In addition, skatole is one of the components of a synthetic pheromone fishers use to attract fish^46^. In a similar way, myristic acid induces landing behaviour in *Culex nigrigalpus* mosquitoes^47^. With all of these, it makes sense to suggest that skatole and myristic acid could be acting as a multicomponent maternal signal attracting piglet to maternal faeces and potentially inducing coprophagy.

Believing that skatole and myristic acid could be acting as faecal maternal semiochemicals (FMS), the effects of spraying skatole and myristic acid on the weaning environment were assessed. Spraying weaned pig feeders with FMS reduced the percentage of time piglets engaged in aggressive behaviours the first 24 h post-weaning by 28% and by 24% over the entire 48 h post-weaning period. No difference in aggressive behaviours was observed from the 24–48 h period. A similar reduction in aggressive behaviours was found by Guy *et al*.^48^ and McGlone and Anderson^49^ when pig feeders were sprayed with the pig appeasing pheromone described by Pageat^50^. The pig appeasing pheromone is a mixture of fatty acids secreted by the sebaceous gland of the mammary gland of lactating sows. In addition to maternal semiochemicals, the use of the androstenone (boar pheromone) also reduced aggression in piglets^51,52^.

The application of FMS on pig feeders tended to increase feeding behaviour the first 24 h post-weaning but did not change the apparent feed intake. Contradictory results are found in the literature about how maternal odours influence pig feeding behaviour. Guy *et al*.^48^ did not find any difference in feeding behaviour when the pig appeasing pheromone was applied to the feeder and floor of weaned pig pens. However, McGlone and Anderson^49^ found that the pig appeasing pheromone increased feeding behaviour the first 48 h post-weaning but it did not affect pig apparent feed intake. Disparities between McGlone and Anderson^49^ and Guy *et al*.^48^ results could be due to doses and application procedure differences. A possible explanation of why feeding and aggressive behaviours were not improved the second day post-weaning could be that after 24 h the FMS application frequency was reduced (i.e. every 12 h instead of every four hours). By decreasing the application frequency, it was possible that piglets were not continuously exposed to the semiochemicals and thus its attractant and calming effect was reduced. Another possible explanation to the lack of behavioural differences in the second day could be that piglets habituated to the semiochemicals. By being habituated to the stimuli, its innate response to maternal semiochemicals could be reduced. Further studies are needed to determine the optimal dose and application frequency in order to improve and prolong the behavioural benefits observed at 24 h post-weaning.

The neural or physiological mechanism by which FMS exhibit a calming and attractant effect in piglets is still unknown. Little or no information is available about how pigs perceive skatole. Since skatole is a very volatile compound, it is most likely to be perceived by the main olfactory system. Myristic acid, on the other hand, could be perceived by both the main olfactory system and the accessory olfactory system. Guiraudie *et al*.^53^ identified four olfactory binding proteins that were involved in the perception of the pig appeasing pheromone. Of these four olfactory binding proteins, the Von Ebner’s gland protein, a protein present in the main olfactory system and vomeronasal organ mucosa, showed a strong affinity for fatty acids^53^. Thus, it is possible that myristic acid could be perceived by both olfactory systems. A possible mechanism by which the FMS exhibit a calming effect could be that, when perceived by the vomeronasal organ, a calming stimulus is sent to the amygdala and this stimulus then could inhibit or reduce aggression^54^. The actual mechanism of the calming effect of this and other maternal odours requires further exploration.

Although FMS had a positive effect on pig behaviour, no significant effect was found on growth performance. Performance data showed that FMS could have a negative effect on pig feed efficiency. The Gain:Feed ratio (G:F) was lower for pigs treated with FMS. In this study, G:F was calculated based on feed disappearance and not in actual feed intake. Actual feed intake can be 10 to 30% lower than feed disappearance due to feeder adjustment^55^. Thus it has been reported that, in some cases, difference in G:F could be due to external factors that could be affecting the actual measurements^55^. Since piglets in different treatments were housed in different rooms to prevent odours exposure in the control group, a confounding effect of room could be influencing performance data. Further studies are needed to determine if the negative effect observed in feed efficiency was due to a treatment or an extraneous factor effect. However, our results agree with Guy *et al*.^48^ who found that the pig appeasing pheromone did not improve pig performance.

## Conclusion

Sow faecal nutrient content changed during lactation. Lactating sow faeces had higher true protein content and are rich in other nutrients and minerals that could be beneficial to piglets. Thus, coprophagy could result in the ingestion of nutrients and minerals that might have a positive impact on piglet performance and health. Twenty-six volatile organic compounds were identified in lactating and gestating sow faeces. No specific analyte was identified on each period. After farrowing, skatole and myristic acid faecal concentrations in fresh faeces increased by 2.68 and 1.13 folds respectively. Preference assessments showed that piglets had a preference for a feeder sprayed with a solution containing skatole and myristic acid. No preference was found when skatole and myristic acid were sprayed individually on the feeder. Spraying weaned pig feeders with skatole and myristic acid reduced the incidence of aggressive behaviours and tended to increase feeding behaviour the first 24 h post-weaning by 28% and 35% respectively. No effects on piglet performance was found when feeders where sprayed with skatole and myristic acid. Since skatole faecal concentration is regulated by sexual hormones and myristic acid is a constituent of domestic animal maternal pheromones, we hypothesized that together they might be acting as a multicomponent faecal maternal signal in swine. The use of these semiochemicals could be a novel tool to improve swine welfare since they had a calming effect on piglets, reducing aggression and tending to improve feeding behaviour.

## Methods

### Identification of maternal semiochemicals

Texas Tech University Institutional Animal Care and Use Committee approved all animal handling, feeding, housing, and sampling procedures used in this study (protocol #16105-11). All procedures used in this study were in accordance with corresponding guidelines and regulation for the use of swine in research. Faecal samples were collected from PIC Camborough line sows (N = 8) at the Texas Tech University Swine Research Facility. During gestation, all sows were housed in crates (2 m × 0.6 m) and, ten days before the expected farrowing day, sows were moved to conventional farrowing crates (1.52 m × 2.13 m). Sows were fed 6.8 kg of lactation diet from two weeks before the expected farrowing day until weaning. This was to prevent that differences in faecal volatile profile and nutritional analysis were not due to a change in diet or an increased feed intake during lactation.

Faecal samples were collected from the same animal during gestation and lactation. Faecal samples were collected daily for five consecutive days after a five days adaptation period to the lactation diet. Gestation faecal samples were collected starting at 10 ± 2 days before the expected farrowing day. Lactation faecal samples were collected for five consecutive days after sow farrowed. Since the first two days after farrowing sows did not have faecal matter for collection, lactation samples were collected from day 3–7 after farrowing. All faecal samples were collected in the morning (0600) directly from the sows’ rectum. Immediately after collection, samples were placed in a whirl-pack bag (Sigma-Aldrich, U.S.A), preserved in liquid nitrogen and transported to the lab. Once in the lab, frozen samples were pulverized, and aliquots of 2 g were prepared for liquid extraction and nutritional analysis. Samples and aliquots were stored at −80 °C until analysis. Liquid extraction was performed within two weeks after collection to prevent significant volatile loss^56^.

Nutritional analyses of gestation and lactation faecal samples were conducted as a composite of the five samples collected in each period. Composites were dried for 48 h at 55 °C, ground and sent by mail to Dairy One, Inc., (Ithaca, NY) for nutritional analyses. The concentration of true protein (TP) in faeces was measured using the bicinchoninic acid (BCA) assay kit (Thermo Fisher Scientific, Bellefonte, PA) following manufacture indications and Paul *et al*.^57^ protein extraction procedure. TP was defined as the amount of biological protein present in sow faeces that reacted to the BCA assay. This differed from the crude protein (CP) in that it does not take in consideration non-protein nitrogen.

The method employed to extract faecal volatiles was an adaptation of Beehner *et al*.^58^ and Rideout *et al*.^26^. The two gram aliquots were placed in a 15 mL falcon tube and mixed with 4 mL of methanol (100%) and1 mL of a 500 µg/ml heptanoic acid solution as internal standard. Subsequently, samples were vortexed at high speed for two minutes and centrifuged at 3,000 rpm for 10 minutes at 15 °C. Once centrifuged, 3 mL of the supernatant was filtered with a polytetrafluoroethylene (PTFE) 0.2 µm syringe filter and diluted in 100 mL of distilled water. After dilution, samples were pre-separated on a previously conditioned reversed phase HyperSep C18 SPE cartridge (2g bed weight; 40–60 µm particle size; 60 Å pore size; 15 mL column capacity; Thermo Fisher Scientific, Bellefonte, PA). Cartridges were conditioned with 10 mL of 100% methanol followed by 10 ml of distilled water. After conditioning, the diluted sample (103 mL) was gradually loaded and filtered at a steady rate (~0.25 mL/sec) using a 12-valve vacuum manifold. After filtration, cartridges were washed with 10 mL of methanol: water solution (1:10 v/v). Filtered and washed material was discarded and analytes were eluted from the cartridge using 3 mL of tert-butyl methyl ether. Eluted material was centrifuged at 3,000 rpm for five minutes to separate the organic solvent from the aqueous layer. Subsequently, 300 µL of the organic solvent was transferred to a screw capped GC-vial.

A GC-MS (Thermo Trace GC-MS Ultra CA; Split/Splitless injection with ISQ Quadrupole Mass Spectrometry detector, Thermo Fisher Scientific Inc., San Jose, CA) was used to analyse faecal sample extracts. The instrument was equipped with an SPB-PUFA capillary column (30 m length × 0.25 mm i.d.; film thickness 0.20 μm; Sigma-Aldrich, U.S.A) with bonded polyalkylene glycol stationary phase. Ultra-high purity helium was used as the carrier gas after it passed through a purification tramp at a flow rate of 1.2 mL/min with vacuum compensation. One micro-liter of the sample was injected by an auto sampler in the injection port in split-less mode. At the time of injection, inlet temperature was 250 °C. Initial oven temperature was set at 140 °C and held constant for 2.5 minutes. Oven temperature was increased at a rate of 4 °C/min until 210 °C and held constant for 35 minutes. The mass spectrometer ion source temperature was 225 °C during analysis and was operating on electron impact mode (70 eV) with scanning range of 45–450 amu. Compounds of interest were identified by comparing the obtained mass spectra from a total ion chromatogram with the instrument control software reference library. The identity of major peaks was further confirmed by comparing the mass spectra and retention time of analytical standards run under the same condition as the samples. Candidate semiochemicals concentrations were estimated using a 6-point calibration curve of the analytical standard at known concentrations. A regression line was calculated for each semiochemical and used to estimate its concentration in the samples.

The five gestation and lactation faecal samples were analysed by GC/MS individually. The instrument software (XcaliburTM, Thermo Fisher Scientific) calculated the area under each peak in the chromatogram. Each peak area was divided by the peak area of the internal standard (heptanoic acid) to obtain an area ratio. Data were analysed using SAS statistical software. For all variables, a normal distribution was confirmed using PROC Univariate. An exploratory analysis was conducted including sow as a block and collection day as a fixed effect. Since no statistically significant difference was found on the peak area ratio within periods (i.e. lactation or gestation), an average ratio was obtained for both periods (gestation and lactation) by averaging the area ratio of the five samples in each period. A paired sample *t-test* was used to evaluate significant differences in peak area ratio, analyte estimated concentration, and nutritional data between periods.

### Preference test

Piglets used in this study were from a terminal cross between PIC Camborough genetic line sows and a terminal sire. Litters were housed in conventional slatted floor farrowing crates (1.52 m × 2.13 m). All piglets were ear notched, teeth clipped, and injected with iron at 3 days of age. During this day, all males within a litter were also castrated by farm personnel. Prior to the study, piglets had no creep feeder. The preference test was performed in their home farrowing crate. Preference tests were conducted to determine piglet preference for skatole and myristic acid. The method used was an adaptation of the one described by Arnould *et al*.^59^ and Osada *et al*.^60^. First, nine litters (105 piglets) were used to evaluate piglet preference toward a solution containing both, skatole and myristic acid (N = 9). Once piglet preference for a solution containing both molecules was determined, 8 litters (79 piglets) were used to evaluate piglet preference for skatole (N = 8) and another 8 litters (80 piglets) to evaluate piglet preference for myristic acid (N = 8) individually. Each litter was tested only once to ensure that piglets were tested at the same age and that they were not previously exposed to a feeder or a semiochemical.

**Figure 4:**
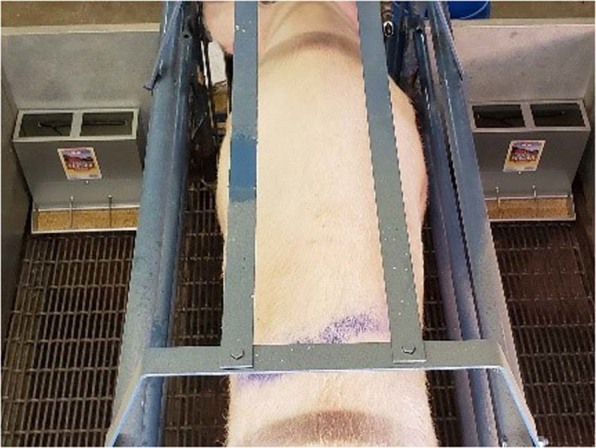
Preference test setting. The two feeders were placed in front of the crate and the sow was facing the feeders. Sow was with the litter during the five-hour testing period.

At 7 ± 2 days of age, two three-hole galvanized steel baby pig feeders were placed at each front corner of the farrowing crate for five hours for two consecutive days (Fig. 4). All trials were performed in the morning when piglets were more active. The sow was present during each five hours trial. Each feeder contained 100 g of pig pre-starter (MoorMan’s ShowTec Prestarter, ADM, Quincy, IL) and was sprayed with either 5 mL of mineral oil (control) or 5 mL of the testing solution. The solution was sprayed on the feeder and not the feed. The 5 mL of the testing solution contained 4.5 mg of skatole and/or 9.0 mg of myristic acid. This concentration was selected based on the estimated concentration of each analyte in 150 g of lactating sow faeces based on GC/MS analysis. On the second testing day, feeder locations were switched to correct for side biases. A single observer following a 30 second scan sampling method, observed the behaviour of each piglet in the litter at 30 second intervals to determine the percentage of time piglets spent interacting with each feeder during the five-hour period each testing day. A total of 600 observations were recorded per litter in each testing day. The percentage of time piglets within a litter spent interacting with each feeder was calculated by dividing the number of time piglets were observed interacting with a feeder over the total number of observations. Interacting with the feeder was defined as when piglets were sniffing, touching or licking the area of the feeder sprayed with the solution or eating or rooting the feed. A preference index was calculated for each feeder by dividing the amount of time piglets were observed interacting with a feeder by the sum of the number of times piglets were observed interacting with both feeders^2^.

For each litter (experimental unit), an overall feed intake, preference index, and percentage of time interacted with each feeder was obtained from both testing days. Difference in overall feed intake, preference index, and percentage of time piglets spent interacting with the control and the feeder sprayed with the testing solutions were analysed using the *Wilcoxon-singed ranked test*. A preference was determined if piglets ate and/or interacted significantly more time with the feeder sprayed with the testing solution. An aversion towards the testing solutions was determined if piglets ate and/or interacted significantly less time with the feeder sprayed with the testing solution. All statistical analyses were performed using SAS 9.4. (SAS Inst., Inc., Cary, NC). A statistically significant difference was based on a *P* value ≤ 0.05.

### Weaning trial

Ninety-six piglets (48 borrows and 48 gilts) from a terminal cross between PIC Camborough genetic line sows and a terminal sire were randomly selected from 14 litters. At 25 ± 2 d of age, piglets were weighed, blocked by weight [heavy 8.4 ± 0.14 kg (32 pigs), medium 7.31 ± 0.08 kg (32 pigs), and light 6.26 ± 0.11 kg (32 pigs)], weaned, and randomly assigned (within block) to be treated with either mineral oil (control) or a solution containing skatole and myristic acid as maternal semiochemicals (FMS). Pigs where blocked by weight to reduce size variation within a pen. Pigs were housed in groups of four (i.e. 2 barrows and 2 females) non-littermate pigs per pen (24 pens total). Raised pens measuring 1.5 × 1.5 m with slat plastic floors were used in this study. Control and FMS treated animals were housed in separate rooms to prevent cross-contamination with the odour between treatments. Piglets had ad libitum access to feed and water and were fed a corn-soybean based diet that met nursing piglet nutrient requirements (NRC, 2012). Control pigs were exposed to mineral oil whereas the FMS treated pigs were exposed to 4.5 mg of skatole and 9.0 mg of myristic acid diluted in five millilitres of mineral oil as in the preference test. Five millilitres of the FMS or mineral oil were sprayed on the feeder (not the feed) of each pen^49^ after the weaning was concluded. Treatments were reapplied on the feeders every four hours until 12 h post-weaning. After 12 h post-weaning, feeders were sprayed every 12 h until 48 h.

All pens (i.e. 12 control and 12 FMS) were video recorded for the first 48 h post-weaning. A single observer evaluated both group feeding and aggressive behaviours from 0–24 hours and from 24–48 hours post-weaning using a scan sample technique at one-minute intervals. A total of 2880 observations were recorded from each pen during the 48-hour period. The percentage of time piglets within a pen spent feeding or displaying aggressive behaviours was calculated by dividing the number of times piglets within a pen were observed doing one of these behaviours over the total number of observations. Aggressive behaviours were defined as when two or more piglets were biting or pushing each other^61,62^. Feeding behaviour was defined as when piglet’s head was inside the feeder trough.

Piglet average daily feed intake, average daily weight gain, and Gain:Feed ratio were measured weekly for four weeks to determine any treatment effect on piglet performance. Average daily feed intake was calculated by estimating weekly feed consumption by weighing the remaining feed on the feeder. Average daily gain was calculated by weekly changes in piglet body weight. Gain:Feed ratio was the ratio between average daily gain and average daily feed intake.

For all statistical analyses the pen was considered the experimental unit (n = 12). Statistical analyses were conducted using SAS 9.4 statistical software. All behaviour and performance data were tested for normality and homoscedasticity. Due to the lack of normality in aggressive behaviour data, its’ statistical analysis was conducted on the inversed transformed data. The back transformed data was reported. Performance and behavioural data were analysed as a complete randomized block design using repeated measures analysis of variance (ANOVA). The model included the effect of time, treatment, and their interaction as fixed effects and pen and weight block as a random effect. There was no treatment by block interaction. Tukey Kramer post hoc test was used for multiple comparisons. A statistically significant difference was based on a *P* value ≤ 0.05.

## Supplementary information

Supplementary information

## Data Availability

Data can be found in the supplementary materials.
