# Supplementary material for: Identification of Faecal Maternal Semiochemicals in Swine (Sus scrofa) and their Effects on Weaned Piglets
Source: Sci Rep. 2020 Mar 24;10:5349. doi: 10.1038/s41598-020-62280-9 (PMC7093430; doi:10.1038/s41598-020-62280-9)
Supplement: Supplementary file 1 — Supplementary information [file 41598_2020_62280_MOESM1_ESM.docx]

IDENTIFICATION OF FAECAL MATERNAL SEMIOCHEMICALS IN SWINE (*Sus scrofa*) AND THEIR EFFECTS ON WEAN PIGLETS

EDGAR AVILES-ROSA^1^, KAZ SUROWIEC^2^, AND JOHN MCGLONE^1*^

^1^ LABORATORY OF ANIMAL BEHAVIOR, PHYSIOLOGY AND WELFARE, *DEPARTMENT OF ANIMAL AND FOOD SCIENCE, COLLAGE OF AGRICULTURE SCIENCE AND NATURAL RESOURSE, TEXAS TECH UNIVERSITY, 1248 INDIANA AVE, LUBBOCK TX, 79415*

^2^*DEPARTMENT OF CHEMISTRY AND BIOCHEMISTRY, COLLAGE OF ART AND SCIENCE, TEXAS TECH UNIVERSTITY, 2500 BROADWAY, LUBBOCK TX, 79409*

EDGAR AVILES-ROSA, [edgar.aviles-rosa@ttu.edu](mailto:edgar.aviles-rosa@ttu.edu)

KAZ SUROWIEC, [Kaz.Surowiec@ttu.edu](mailto:Kaz.Surowiec@ttu.edu)

^*^JOHN MCGLONE, [john.mcglone@ttu.edu](mailto:john.mcglone@ttu.edu)

**Supplementary Material**

Table 1 Behaviour definitions

| Behaviours |  |
| --- | --- |
| Feeder interaction | Piglets were sniffing, touching or licking the area of the feeder sprayed with the solution or eating or rooting the feed |
| Aggressive Behaviours | Two or more piglets were biting or pushing each other |
| Feeding | Piglet’s head was inside the feeder’s trough |
